# TRIM for Tissue Specificity

**DOI:** 10.1021/acsmedchemlett.3c00504

**Published:** 2023-12-26

**Authors:** Marcus D. Hartmann

**Affiliations:** Department of Protein Evolution, Max Planck Institute for Biology Tübingen, 72076 Tübingen, Germany; Interfaculty Institute of Biochemistry, University of Tübingen, 72076 Tübingen, Germany

## Abstract

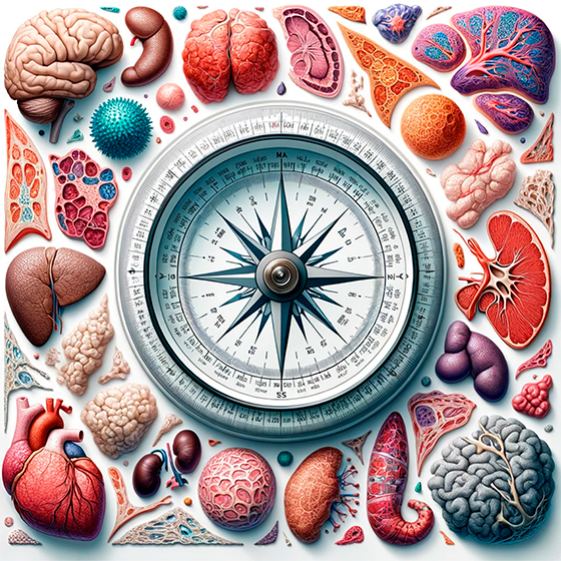

Tissue-specific manipulation of proteins is a long-standing objective in the field of targeted protein degradation, but still a distant prospect. Currently, the most successfully employed E3 ubiquitin ligases belong to the most ubiquitously expressed representatives. Unlocking of the TRIM58 ligase might represent a promising step toward tissue-specific PROTACs and molecular glue degraders.

Targeted Protein Degradation (TPD) via the ubiquitin-proteasome pathway is an omnipresent topic in the biomedical literature, with new Molecular Glue (MG) degraders and Proteolysis Targeting Chimeras (PROTACs) being reported in an inflationary manner. Both MGs and PROTACs are proximity-inducing modalities that recruit a Protein Of Interest (POI) to a specific E3 ubiquitin ligase for its ubiquitination and subsequent proteasomal degradation. However, despite a tremendous repertoire of many hundred different E3 ligases that could potentially be employed for TPD purposes, we rarely see reports on new approaches that utilize ligases other than the handful of already established representatives, which are mostly expressed ubiquitously throughout our tissues. For many reasons, it is important to expand the set of ligases that are currently in our TPD toolkit. One of the most prominent ones is to be able to employ ligases that are exclusively expressed in a specific tissue. Scientists from Novartis now describe their quest on identifying and characterizing ligands for TRIM58, a ligase that is exclusively expressed in late-stage erythroblasts and implied in erythrocyte development.^1^ Their study not only provides a potential starting point for the possible development of erythroblast-specific protein degraders, but also exemplifies a state-of-the-art drug discovery campaign in industry.

The more than 600 identified human E3 ubiquitin ligases differ tremendously in their physiological roles. They are specific for the recognition of different substrates, specific for the transfer of either ubiquitin or a ubiquitin-like modifier, and specific for the type of linkage connecting the modifier(s), which in turn may be specific for proteasomal degradation but also for many other regulatory purposes. Moreover, E3 ubiquitin ligases differ in their spatiotemporal expression levels; they may be specific for certain tissues, developmental stages, but also for certain disease contexts and cancer types. While E3 ligases that are ubiquitously expressed in all tissues could be utilized for TPD in essentially any context, a restriction to a specific tissue would be highly desirable in many applications. TPD is still an emerging modality with particular safety concerns. The best-studied degraders are thalidomide and its FDA-approved derivatives, which act as MGs via the ubiquitously expressed ligase CRL4^CRBN^. However, via this mode of action, thalidomide also addresses at least one off-target responsible for its notorious teratogenic effects.^2^ Newer thalidomide derivatives have an improved POI selectivity profile and are therefore thought to be less potent teratogens. Nevertheless, by controlling not only POI selectivity but also spatiotemporal specificity, the employment of tissue-specific E3 ligases could greatly contribute to generally enhanced safety profiles of future PROTACs or MG degraders.

As much as the hundreds of human E3 ligases differ in their physiological roles and expression patterns, they also differ in their structural architectures and domain composition. The majority of the ligases currently employed in TPD, including CRL4^CRBN^, belong to the RING ligases, specifically the class of cullin-RING ligases. RING ligases are a heterogeneous group comprising both the complex, muti-subunit cullin-RING ligases, as well as simple, single-polypeptide RING ligases. With more than 80 identified members, the tripartite motif (TRIM) family proteins represents the largest group of these simple RING ligases. The individual members of this family are implied in different disorders, and many are selectively expressed in disease-relevant tissues.^3^ For recognizing their substrates, they employ different domains and domain architectures, which is reflected by a further classification into several subfamilies. However, about half the identified TRIM ligases—those in subfamilies I and IV—employ a C-terminal SPRY or PRY-SPRY domain for substrate recognition.^4^

In their present report, the Novartis team was tackling the substrate recognition mode and identifying ligands for the PRY-SPRY domain of TRIM58. This ligase is exclusively expressed in late-stage erythroblasts and known to be responsible for the degradation of multiple targets, including the dynein complex during the enucleation step of erythroblast development.^5^ They identified first hits in a small-molecule library screen based on Differential Scanning Fluorimetry (DSF), a method that requires little assay development and can be performed without prior functional knowledge on the target protein. In this assay, compound TRIM-473 was identified as the front-runner, which was further validated in protein-observed NMR and SPR experiments. In a next step, the team designed a fluorescent reporter peptide to perform competitive Fluorescence Polarization (FP) displacement assays for the further development of TRIM-473. For this, they exploited the sequence of the known TRIM58 binder Dynein Intermediate Chain (DIC) as well as first structural insights gained from a putative crystallographic artifact in an apo TRIM58 structure that they determined. Further, they determined the crystal structure of the TRIM58::TRIM-473 complex, to inform a Structure Activity Relationship (SAR) study for the improvement of TRIM-473. Although this SAR, which was quantified by the FP assay, did not yield further improvements of TRIM-473, the whole study showcases the possible potential of TRIM58 and maybe also other TRIM subfamily I and IV members in TPD.

PRY-SPRY domains generally utilize a rather shallow binding interface for the recognition of their target proteins.^6,7^ As already suggested by the FP-based displacement assay, the TRIM58::TRIM-473 complex structure revealed that TRIM-473 also binds to the canonical interaction interface. While the shallow character of this interface makes ligand improvement difficult, it offers a variety of possibilities for modifications and substitutions on TRIM-473 without impacting binding affinity. This could be exploited for designing different exit vectors for PROTAC candidates, but conceivably also for the development of MG degraders that modulate the PRY-SPRY domain substrate recognition interface. Although there might be room for affinity improvement, it should be noted that there are prominent examples of MG degraders that have surprisingly poor affinity for their dedicated ligase in absence of the POI.^8^ Consequently, taking into account the similarity of their individual PRY-SPRY domains, we might be looking not only at the prospective TPD-enabling erythroblast-specific ligase TRIM58, but also at diverse members of two TRIM subfamilies with the potential to be unlocked for tissue-specific TPD via TRIM-473-inspired PROTACs or MG degraders.
